# Subject-specific multiscale modeling of aortic valve biomechanics

**DOI:** 10.1007/s10237-021-01429-5

**Published:** 2021-04-01

**Authors:** G. Rossini, A. Caimi, A. Redaelli, E. Votta

**Affiliations:** Departement of Electronics, Information and Bioengineering, Politecnico di Milano, Via Ponzio 34/5, 20133 Milan, Italy

**Keywords:** Aortic valve, Valve interstitial cells, Multiscale approach, Subject-specific simulations, Finite element modeling

## Abstract

A Finite Element workflow for the multiscale analysis of the aortic valve biomechanics was developed and applied to three physiological anatomies with the aim of describing the aortic valve interstitial cells biomechanical milieu in physiological conditions, capturing the effect of subject-specific and leaflet-specific anatomical features from the organ down to the cell scale. A mixed approach was used to transfer organ-scale information down to the cell-scale. Displacement data from the organ model were used to impose kinematic boundary conditions to the tissue model, while stress data from the latter were used to impose loading boundary conditions to the cell level. Peak of radial leaflet strains was correlated with leaflet extent variability at the organ scale, while circumferential leaflet strains varied over a narrow range of values regardless of leaflet extent. The dependency of leaflet biomechanics on the leaflet-specific anatomy observed at the organ length-scale is reflected, and to some extent emphasized, into the results obtained at the lower length-scales. At the tissue length-scale, the peak diastolic circumferential and radial stresses computed in the fibrosa correlated with the leaflet surface area. At the cell length-scale, the difference between the strains in two main directions, and between the respective relationships with the specific leaflet anatomy, was even more evident; cell strains in the radial direction varied over a relatively wide range ($0.36-0.87$) with a strong correlation with the organ length-scale radial strain ($R^{2}= 0.95$); conversely, circumferential cell strains spanned a very narrow range ($0.75-0.88$) showing no correlation with the circumferential strain at the organ level ($R^{2}= 0.02$). Within the proposed simulation framework, being able to account for the actual anatomical features of the aortic valve leaflets allowed to gain insight into their effect on the structural mechanics of the leaflets at all length-scales, down to the cell scale.

## Introduction

The aortic root (AR) is the bulb-like functional and anatomical unit connecting the outlet of the left ventricle to the ascending aorta. The aortic valve (AV) is the central structure in the AR; it is normally composed of three leaflets that, in physiologic conditions, open during systolic ejection from the ventricle into the aorta and close during ventricular diastole. The leaflets are characterized by a three-layered structure: the fibrosa layer faces toward the aorta and is rich in type I collagen fibers preferentially oriented in the circumferential direction, i.e., from commissure to commissure; the ventricularis layer faces toward the left ventricle and is mainly composed of elastin fibers preferentially oriented in the radial direction, i.e., from the leaflet attachment line onto the aortic wall to the leaflet free edge; the spongiosa layer separates the fibrosa and the ventricularis and is composed of proteoglycans (PGs)/glycosaminoglycans (GAGs) Brazile et al. (2015). The different microstructure of the fibrosa and ventricularis reflects into their markedly different stress-strain response as characterized by means of biaxial tensile tests Stella and Sacks (2007) Sacks and Yoganathan (2007), which in turn determine the nonlinear and anisotropic mechanical properties of AV leaflets Billiar and Sacks (2000). The fibrosa and the ventricularis are populated by valve interstitial cells (VICs), which regulate the homeostasis of tissue microstructure Taylor et al. (2003). Of note, the cyclic time-dependent pressure loads acting on the AV leaflets are transmitted to the tissue layers and finally down to the cell scale and influence the behavior of VICs. Variations in these physiological macroscopic mechanical stimuli may alter VICs’ matrix-maintaining role and lead to pathological tissue remodeling, as in calcific AV Otto (2002), characterized by formation and growth of calcific noduli in the fibrosa layer Aikawa and Libby (2017). This evidence suggests for the fibrosa a leading role in AV remodeling processes, despite the presence of VICs in both the fibrosa and ventricularis layers, advising the need for the investigation of the stress-strain distribution within the leaflet thickness during the cardiac cycle. The understanding of remodeling-driven pathologies in the AV and in the other heart valves is of strong clinical interest and has been motivating a growing body of research investigating the mechanism of stimuli transmission from the organ to the cell scale Lee et al. (2015) Bakhaty et al. (2017). Of note, the experimental quantification of the mechanical properties of VICs and of the VIC-matrix, which influence this transmission, is still a challenge; only micropipette aspiration estimations of the elastic modulus of VICs are available David Merryman et al. (2005). In this context, finite elements (FE) simulations may represent an alternative way to bypass this limitation. These have been largely used for the simulation of AV behavior at different scales Labrosse et al. (2010), Bakhaty et al. (2017), Sakamoto et al. (2017). However, despite the simulation of AV biomechanics at different scales has been deeply investigated separately, to the best of our knowledge few works tackled the multiscale relationship among the different scales Weinberg and Mofrad (2007), Weinberg et al. (2010). Understanding the role of biomechanics in the complex remodeling pathways would be greatly enhanced by a framework linking AV macroscopic strains and stresses to the mechanical stimuli experienced by VICs; moreover, these stimuli might be successfully used as input conditions in tissue engineering experimental setups embedding VICs. To do so, it is mandatory to obtain a range of values which can be considered physiological, to be used as term of comparison for the range of stimuli related to a specific pathological condition (e.g., bicuspid aortic valve or BAV). Taking into account the inter-leaflets variability of a subject-specific model is therefore mandatory to obtain such a range of values, which would be impossible to capture using a general paradigmatic model with standard geometry. Multiscale FE simulations have been explored in literature to investigate tissue responses in different biomechanical fields other than heart valves, such as in bone mechanics as reported by Johnson and Troy (2017), and Vaughan et al Vaughan et al. (2012). Moreover, different mathematical formulations are exploited to describe the multiscale biomechanical response of biological tissues (e.g., Marino and Vairo (2012) on their study on collagen bio-structures and Pastrama et al. (2018) on bone remodeling). The-state-of-the-art in the context of multiscale AV FE simulations is represented by the work by Weinberg and Mofrad (2007). This work used a fully three-dimensional AV geometry, including appropriate nonlinear, anisotropic material models; at the organ-scale a dynamic fluid-structure interaction (FSI) model predicted the full cycle of AV opening and closure. The tissue-scale model simulated the behavior of AV cusp tissue multiple layers. The cell-scale model computed deformations of individual cells within the cusps. Each cell-scale model was fed by displacement boundary conditions obtained from the immediately higher length-scale model. Despite representing a solid multiscale approach, that workflow has been tested on a single organ scale anatomy which is paradigmatic and symmetrical, preventing from considerations related to the leaflet-specific anatomical features of the model and thus hampering the possibility to assess, if any, the dependency of cell mechanical stimuli on leaflet geometry. With the aim of overcoming this limitation in the present work, we propose a FE multiscale workflow that computes AV biomechanics at the organ length-scale throughout the cardiac cycle based on subject-specific anatomies, thus capturing inter-leaflet differences related to asymmetries as well as inter-subject variability. Moreover, the workflow transfers the organ-scale information down to the cell length-scale exploiting different boundary conditions approaches: displacement boundary conditions are used to transfer the data from the organ to the tissue length scale, while stress boundary conditions are passed from the tissue to the cell scale. The workflow was applied to three subject-specific AV anatomies with the aim of testing one main working hypothesis: patient specific models and different boundary conditions approaches improve the prediction of VICs strain and discriminate among the different leaflet layer role. In this context, the designed modeling approach allows to capture subject-specific and leaflet-specific features in terms of difference in the strain patterns at the organ scale and to transfer this variability down to the cell scale, leading to a range of strain values describing the VICs mechanical response.

## Materials and methods

### Multiscale workflow

**Fig. 1:**
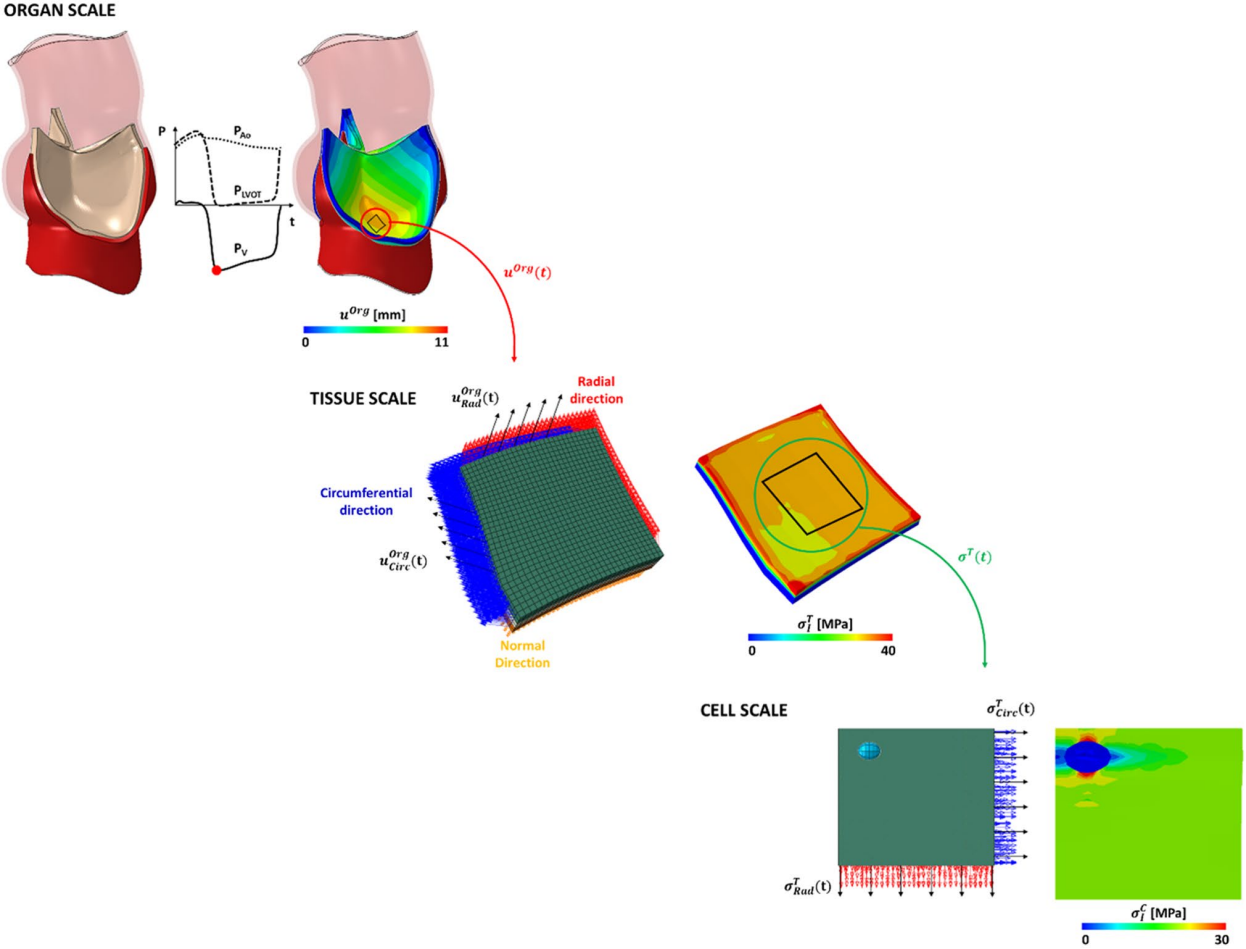
AR Biomechanics is simulated over two cardiac cycles; a leaflet subregion (black square evidenced by a red circle) is considered and the time dependent nodal displacement ($\overrightarrow{{u}^{{org}}}$ ) at the boundary of the subregion are extracted. Tissue length scale. The leaflet subregion is considered, and the layered structure of the tissue is accounted for (green = fibrosa, white = spongiosa, orange = ventricularis). The displacement boundary conditions ($\overrightarrow{u}^{{org}}_{Rad}$, $\overrightarrow{u}^{{org}}_{Circ}$) oriented to the fiber directions are used as boundary conditions and the time dependent stresses ($\overrightarrow{\sigma}^{{T}}$) are extracted from the fibrosa layer, away from the boundaries. The fibrosa layer is seeded with one VIC (highlighted in cyan), the stresses $\overrightarrow{\sigma}^{{T}}_{Rad}$ and $\overrightarrow{\sigma}^{{T}}_{Circ}$are used as boundary conditions and the time dependents cell stretches are computed

The modeling workflow consists of three simulations at three different length-scales: organ (*m* to *cm*), tissue (*mm*) and cell ($\mu m$). These are organized in a top-down scheme, so that each simulation provides data to feed the simulation at the immediately smaller length-scale, see Fig. 1.

### Organ length-scale model

The organ length-scale model of the AR is based on a refined version of the approach described in Votta et al. (2017) and is here briefly summarized.

**Fig. 2:**
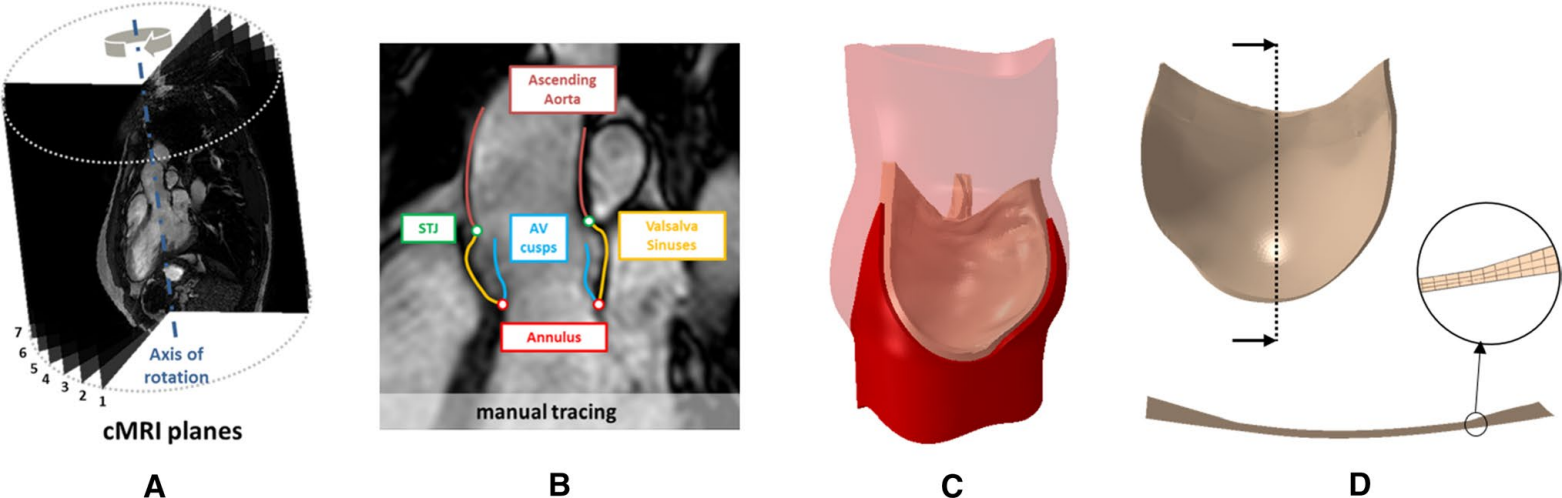
**a** cMRI planes (6 out of 18 planes are depicted). **b** AR substructures manually traced on a cMRI plane. **c** Reconstructed 3D geometry of the AR. **d** Detail of an AV leaflet

*Acquisition of cMRI data and segmentation* Cardiac magnetic resonance imaging (cMRI) was performed on 3 healthy volunteers (Table 1). T1-weighted cine-cMRI sequences were acquired on 18 planes evenly rotated around the axis passing through the center of the annulus and the center of the sino-tubular junction (Fig. 2a). Acquisitions were performed on a 1.5T Achieva scanner (Philips Healthcare Medical System, Irvine, CA, US). In-plane spatial resolution and slice thickness were 1.1 mm and 7 mm, respectively. Thirty frames/cardiac cycle were acquired with R-wave triggering. In the first systolic frame, when the transvalvular pressure acting on AV leaflets was considered negligible Jane Grande et al. (1998), AR substructures were manually traced through in house Matlab (The Mathworks, Inc., Natick, MA, US) scripts (Fig. 2b).

**Table 1:** Table of Volunteers

	Subject 1	Subject 2	Subject 3
Gender [M/F]	M	M	M
Age [years]	28	31	27
Weight [kg]	65	90	90

*Reconstruction and discretization of AR geometry* Manual tracings yielded a point-cloud, which was filtered to eliminate noise effects. A 3D surface for each AR structure was created and discretized with quadrangular shell elements with characteristic dimension of 0.4 mm. From this mesh, a volumetric one was automatically obtained. The aortic wall volumetric mesh was obtained by copying and translating the nodes of the shell elements along the corresponding local outward normal; for each quadrilateral element of the aortic wall surface, three layers of hexahedral solid elements (Abaqus C3D8 elements) were obtained (Fig. 2C). Since aortic wall actual thickness was not assessable from cMRI, a wall thickness of 1.0 mm was assumed Jane Grande et al. (1998), and the 3 layers accounted for $15\%$, $50\%$ and $35\%$ of the total thickness Holzapfel et al. (2005). The leaflets volume mesh was obtained through an ad-hoc extrusion process in order to create a paradigmatic and region-dependent thickness pattern, ranging from 0.4289 mm in the belly region to 1.2 mm at the attachment edge and at the free margin Jane Grande et al. (1998), (Fig. 2D). Leaflets are described as layered, with the fibrosa, the spongiosa and the ventricularis accounting for $41\%$, $30\%$ and $29\%$ of the total thickness Stella and Sacks (2007). The characteristic in-plane mesh size of the elements is approximately 0.4 mm both for the aortic wall and the AV leaflets.

*Tissues mechanical properties* Aortic wall tissue was assumed linear, elastic and isotropic, with a 2 MPa Young modulus and a 0.3 Poisson ratio Conti et al. (2010). AV leaflet tissue was described as anisotropic and hyperelastic, through the strain energy function U of the form:

1 $$\begin{aligned} U = \frac{C}{2}(e^{Q}-1)+K\left( \frac{J^{2}-1}{2}-lnJ\right) \end{aligned}$$

where C is the first constitutive parameter, J the determinant of the deformation gradient tensor **F**, K is the bulk modulus, and Q is derived from the one originally proposed by Guccione et al. (1991):

2 $$\begin{aligned} \begin{aligned} Q =&2b_{1}Tr(E) + b_{2}E_{ff} \\&+ b_{3}(E_{ss}^{2}+E_{nn}^{2}+E_{ns}^{2}+E_{sn}^{2}) \end{aligned} \end{aligned}$$

**Fig. 3:**
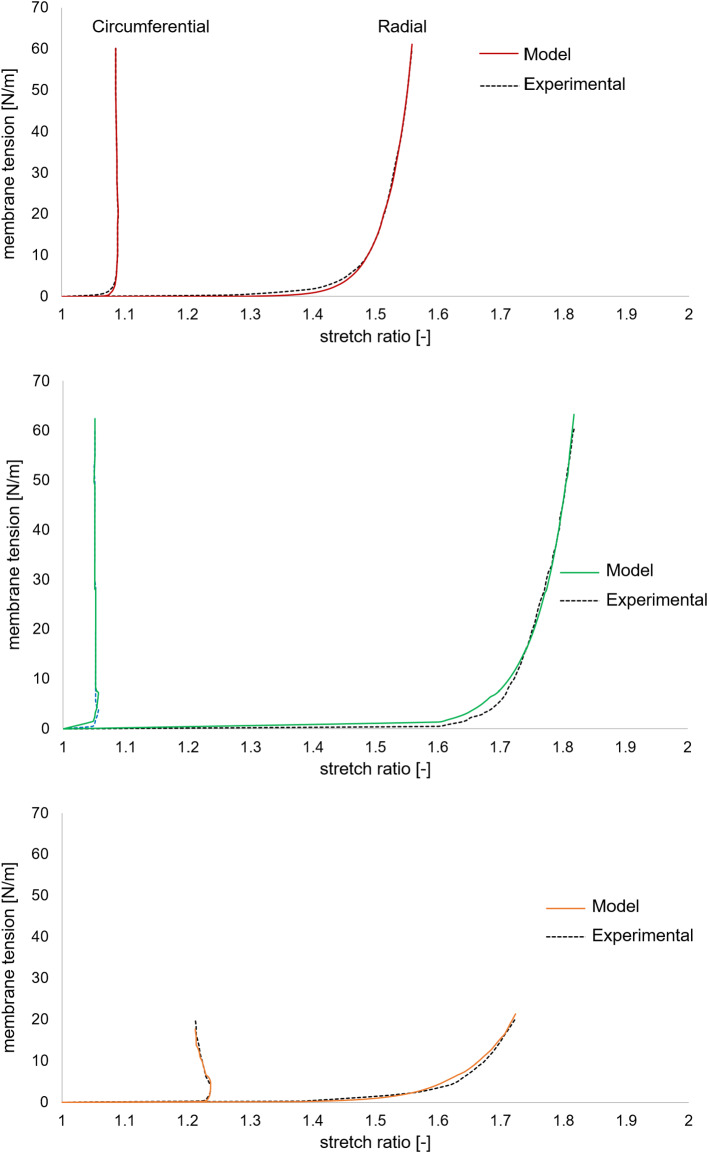
Fitting of experimental data for the intact leaflet (top), fibrosa layer (middle) and ventricularis layer (bottom)

The terms $E_{ij}$ are the components of the Green-Lagrange strain tensor, expressed with reference to a coordinate system *f*, *s*, *n* whose axes are aligned with preferentially oriented fibers (*f*) and orthogonally to them (*n*, *s*). In the model, *f*, *s*, *n* were considered aligned with the commissure-commissure, annulus-to-free margin and through-thickness direction, respectively. *C* and $b_{1}$, $b_{2}$, $b_{3}$ are the constitutive parameters, which were identified by least square fitting of experimental data from biaxial tensile tests by Stella and Sacks (2007), see Fig. 3, and are reported in Table 2. The constitutive model was implemented into a VUANISOHYPER-STRAIN subroutine for the commercial solver ABAQUS Explicit (Dassault Systemes, Providence, RI, USA).

**Table 2:** Table of Fitting

	$C\, [\mathrm{MPa}]$	$b_{1} [-]$	$b_{2} [-]$	$b_{3} [-]$	$K\, [\mathrm{MPa}]$
Intact leaflet	2.82E-03	0.0	12.84	1.97	50
Fibrosa	3.48E-06	5.49	81.48	-0.08	50
Ventricularis	5.09E-06	4.86	5.02	-1.63E-03	50

*Computation of AV biomechanics* The initial configuration of the AR was defined at early systole, when AV leaflets are approximately unloaded, but the aortic wall is pressurized to 80mmHg. Consistently, aortic wall pre-stresses were computed through the iterative process described in Votta et al. (2017). Displacement boundary conditions were imposed to the nodes at the proximal and the distal ends of the AR model, so to allow only radial expansion or contraction of the AR wall. A scale penalty method was used to model mechanical interactions orthogonally to the contacting surfaces, while a 0.05 friction coefficient was set to model tangential contact interaction Votta et al. (2017). The structural response of the pre-stressed AR was computed over two consecutive cardiac cycles; to this aim, physiological time-dependent ventricular and aortic pressures were applied to the aortic wall upstream from and downstream of the AV, respectively, and a consistent trans-valvular pressure drop was applied to the AV leaflets Conti et al. (2010). The circumferential and radial strains were averaged over a patch of $5 \times 5 \times 3$ elements ($\approx$ 2 mm circumferential x $\approx$ 2 mm radial x 0.43 mm normal) in the central part of the belly region which experienced the highest strain values Labrosse et al. (2010).

### Tissue length-scale model

**Fig. 4:**
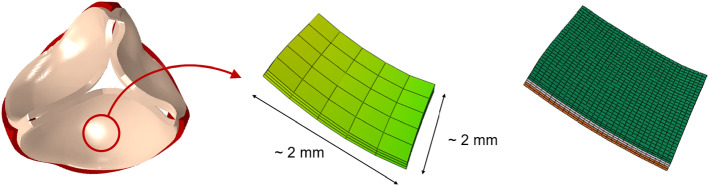
From the left to the right: the organ scale model, the selected patch with the original mesh and the remeshed patch with the layer-specific material definition

Every leaflet patch selected at the organ length-scale was remeshed to refine the discretization while preserving its original shape. Of note, each patch was selected so that the boundary faces of the patch consisted in a set of faces of the original elements. Every element of the organ scale patch (75 elements in total) was divided in 72 elements; to this aim, the position of the new nodes was obtained by interpolating the original nodal coordinates through the shape functions of the corresponding original elements. This procedure yielded a new mesh of 5400 C3D8 elements (characteristic size of the elements $\approx$ 0.06 mm), see Fig. 4.

The three different layers of the AV leaflets were described through a dedicated mechanical characterization; for the fibrosa and the ventricularis layers, we assumed an anisotropic and hyperelastic behavior, using the same formulation as in Equation 1. The model parameters were identified by least square fitting of experimental data from Stella and Sacks (2007) on the AV leaflet layers, as plotted in  Fig. 3 and reported in Table 2. The spongiosa layer was described instead as linear, elastic and isotropic, with an elastic modulus of 0.02 MPa and a Poisson ratio equal to 0.49. The nodal displacements imposed at the boundary of the patch were obtained by interpolating the displacements of the nodes at the boundary of the same region in the organ length-scale model through the shape functions of the corresponding elements. First, the stress-free patch was linearly stretched to the configuration at the beginning of the diastolic phase of the ${2^{\mathrm{nd}}}$ cardiac cycle, and then the displacement curve was applied. Circumferential and radial stress and strain data were then extracted in the central region of the fibrosa layer in order to avoid boundary effects.

### Cell length-scale model

**Fig. 5:**
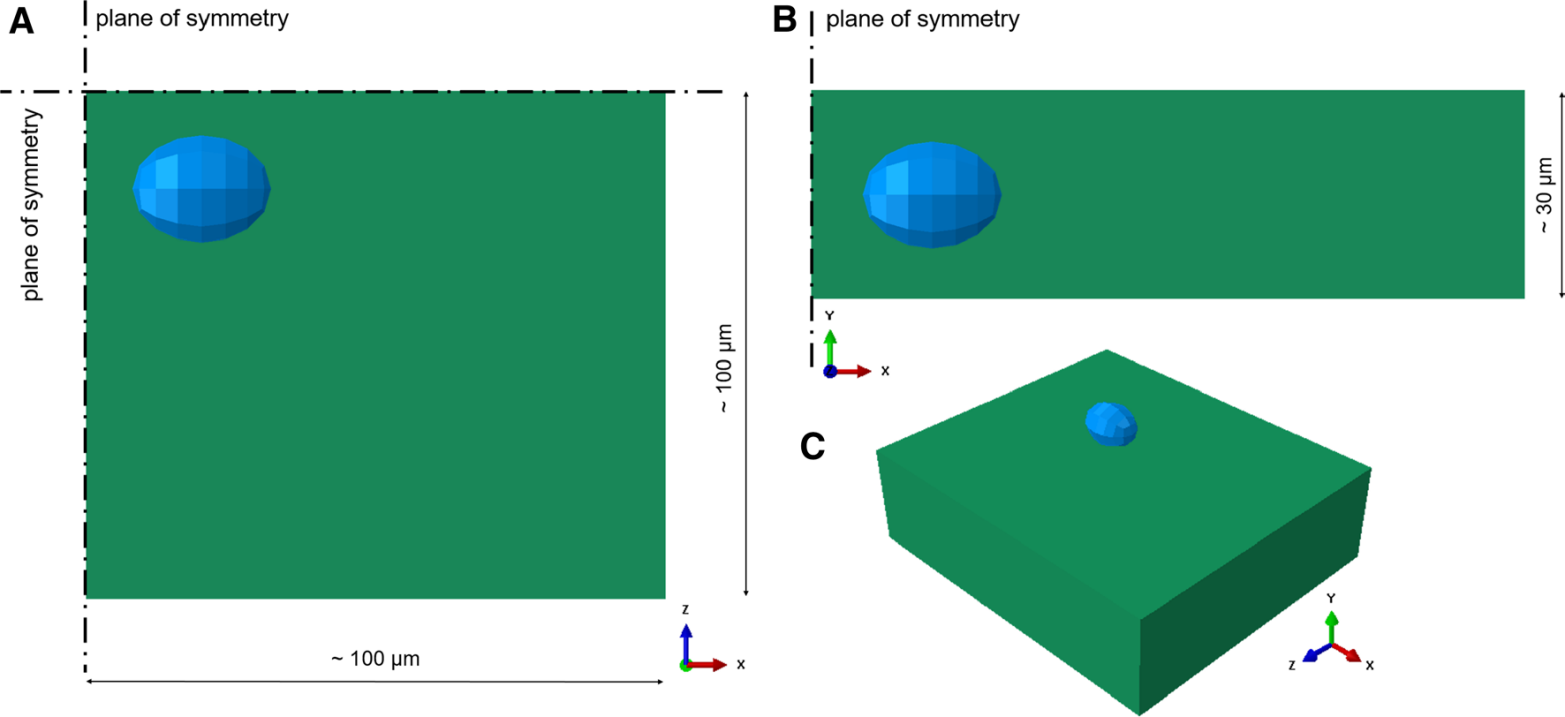
Top (**a**), side (**b**) and isometric (**c**) view of the cell length-scale patch

The cell scale model consists of a patch of fibrosa extra cellular matrix (ECM) with one embedded VIC, characterized by a full mesh continuity between the two subsets of elements. The constitutive model for the fibrosa ECM elements in the cell length-scale patch is the same one used at the tissue length-scale for the fibrosa layer, with the same model parameters reported in Table 2. The elements belonging to the VIC inclusion are instead assigned a linear, elastic and isotropic material model with a Young modulus E = 9E-04 MPa and a Poisson ratio $\nu = 0.45$ Shadow Huang (2004). The patch measured approximately 100 $\upmu \mathrm{m}$ in the circumferential and radial direction and 30 $\upmu \mathrm{m}$ in the normal direction, and cell inclusion was embedded in the corner of the patch. Three planes of symmetry were defined on the computational domain, consisting of approximately 5200 C3D8 elements, with a mesh size of 0.005 mm. Accordingly with experimental measurements from literature Shadow Huang (2004) the axes of the VIC were defined to be 10 $\upmu \mathrm{m}$ in the circumferential direction and 7.7 $\upmu \mathrm{m}$ in the radial and normal directions. Load boundary conditions derived from the tissue scale simulations was applied in the circumferential and radial direction; the stress-free patch was pre-loaded using the stress field at the beginning of diastole, then the tissue-derived stress curves were applied in the radial and circumferential direction, respectively (Fig. 5).

## Results

### Organ length-scale model

**Fig. 6:**
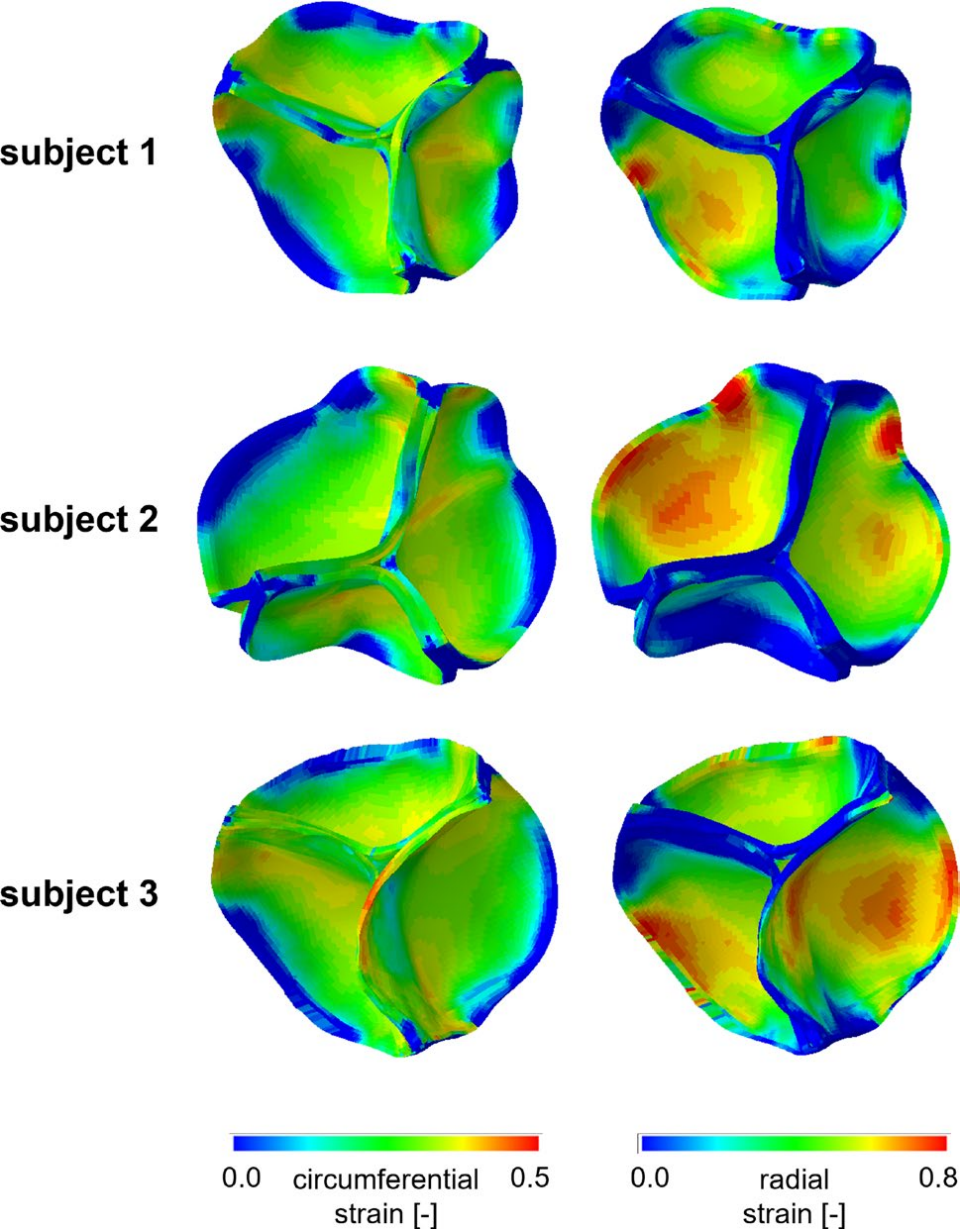
Aortic view of the circumferential (right column) and radial (left column) strain computed on AV leaflets surface at peak diastolic transvalvular pressure for each simulated AV model

**Fig. 7:**
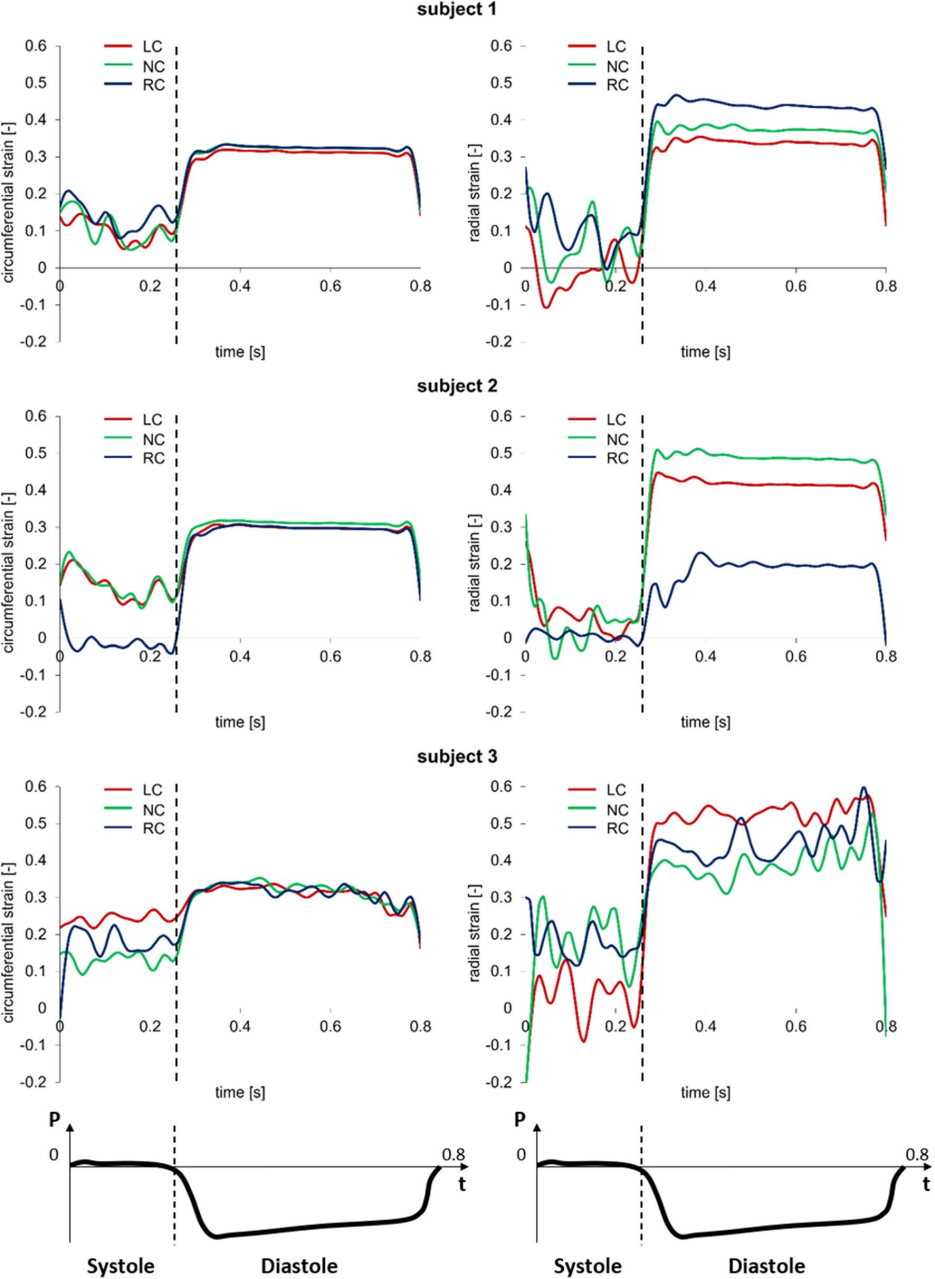
Circumferential (left column) and radial (right column) stretch ratios in the center of the belly region of the left-coronary (LC), non-coronary (NC) and right-coronary (RC) leaflets over the systolic and diastolic phases of the ${2^{\mathrm{nd}}}$ simulated cardiac cycle for the three subjects. The time-course of the transvalvular pressure is reported in the bottom row of each column

The AV biomechanics for the three subjects were evaluated on the second cardiac cycle simulated. First, the space-distribution of leaflet circumferential and radial strains were analyzed at peak diastole, when the strains were expected to be maximal (Fig. 6). The three valves were characterized by differences in the strain distribution; because the same loading conditions, the same tissue mechanical properties, and the same tissue thickness distribution were assumed for each simulated AR, differences in strain distribution at peak diastole were related to anatomical differences in terms of leaflet surface extent and proportions. Despite this variability, the highest strains values were always computed in the belly region, while the attachment edge showed almost no-deformation in both the circumferential and radial direction. To focus the analysis on the most deformed region of the leaflets, at each time point of the cardiac cycle, the radial and circumferential components of the strain tensor were averaged over the extent of the belly region. The corresponding time-courses confirmed that maximal values were obtained at peak diastole, when the peak transvalvular pressure loads the leaflets, just before a plateau extended to almost the entire diastolic phase (Fig. 7).

This behavior is even more clear considering the stretch ratios curves reported in Fig. 7, and looking in particular to the the diastolic phase, i. e. the plateau part of the graphs.

**Fig. 8:**
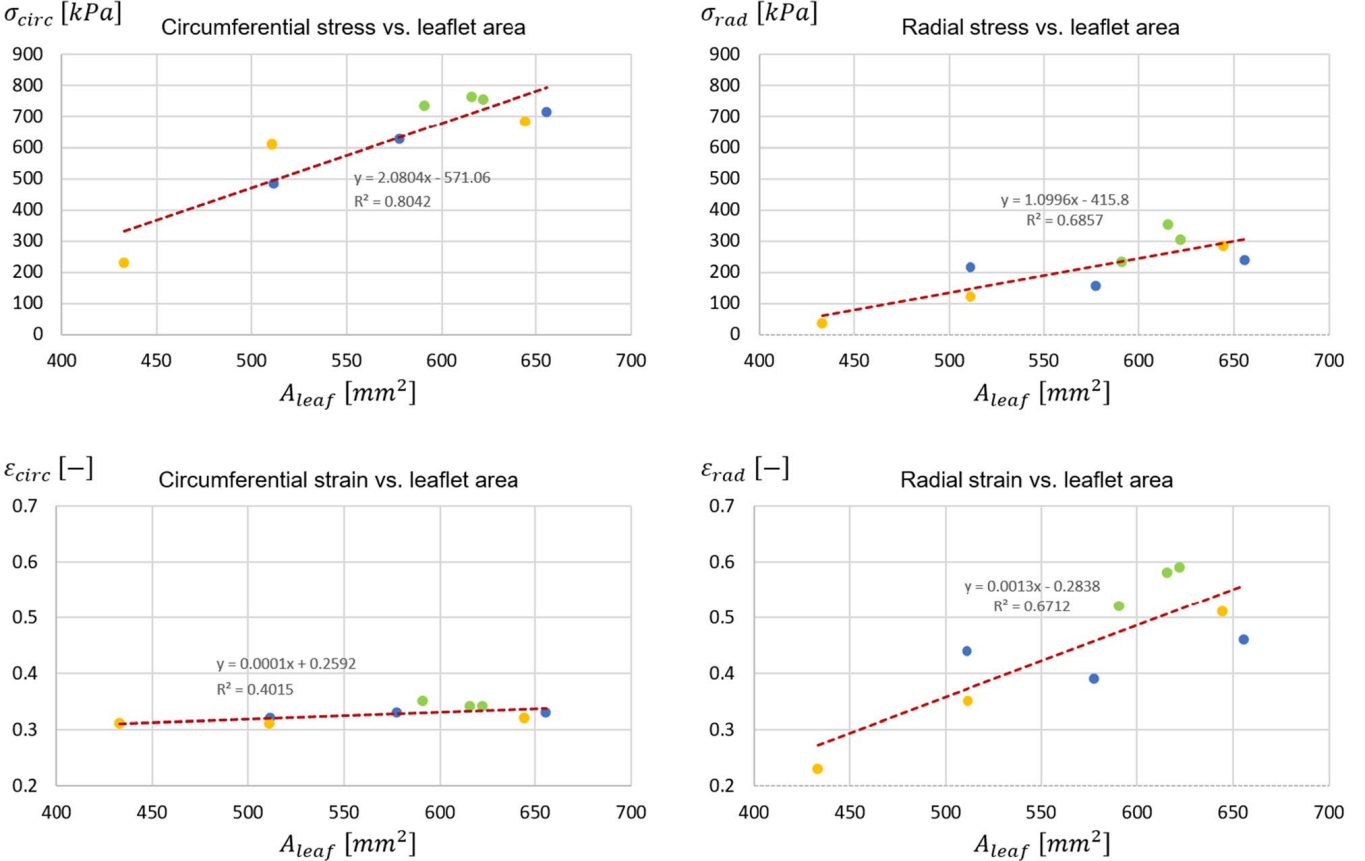
Linear regression between the peak diastolic value of circumferential stress ($\sigma _{circ}$) and radial stress ($\sigma _{rad}$) (top row) vs. leaflet surface area, and between the peak diastolic circumferential strain ($\epsilon _{circ}$) and radial strain ($\epsilon _{rad}$) (bottom row) vs. leaflet surface area. For each stress and strain component, the data represent the maximum value reported in the plots of Fig. 7. Dots = data computed by the models of subject 1 (blue), subject 2 (yellow) and subject 3 (green); dashed red line = regression line. $R^2$ is reported

When comparing different leaflets, whose surface area ranged from 512 ${\mathrm{mm}^{2}}$ to 656 ${\mathrm{mm}^{2}}$ in subject 1, from 433 ${\mathrm{mm}^{2}}$ to 644 ${\mathrm{mm}^{2}}$ in subject 2, and from 590 ${\mathrm{mm}^{2}}$ to 622 ${\mathrm{mm}^{2}}$ in subject 3, peak diastolic radial strains ranged in a broad interval (0.23 - 0.59). A rather clear positive linear correlation (p-value = 0.0068 and a $R^2$ = 0.6712) was found. Instead, peak diastolic circumferential strains ranged in a very narrow range (0.31 - 0.35) and seemed barely influenced by the surface extent of the leaflet (Fig. 8). The same type of analysis was performed on leaflet belly peak diastolic stresses. Both the circumferential and the radial component of the stress tensor increased with the leaflet surface area: the peak diastolic circumferential stress ranged from 230 kPa to 763 kPa, the peak diastolic radial stress ranged from 36 kPa to 352 kPa. For both components, a good positive linear correlation with leaflet surface area was found (p-value equal to 0.0011 and 0.0059, and $R^2$ equal to 0.80 and 0.69 for the circumferential and radial component, respectively). The effect of leaflet surface area on the leaflet belly stresses was more pronounced for the circumferential stress component as compared to the radial one (Fig. 8).

### Tissue length-scale model

**Fig. 9:**
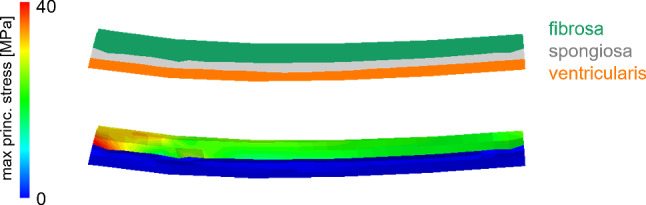
Section of the LC leaflet patch of subject 1; the three different layer are represented on the top, and the max principal stress through the thickness is plotted on the bottom

The max principal stress was analyzed in order to check its distribution through the leaflet thickness. Consistently with their very different mechanical proprieties, the layers experienced very different stress levels, as exemplified in Fig. 9 for the LC leaflet of subject 1. The fibrosa layer showed to bear most of the leaflet tensions as compared to the spongiosa and ventricularis layers.

**Fig. 10:**
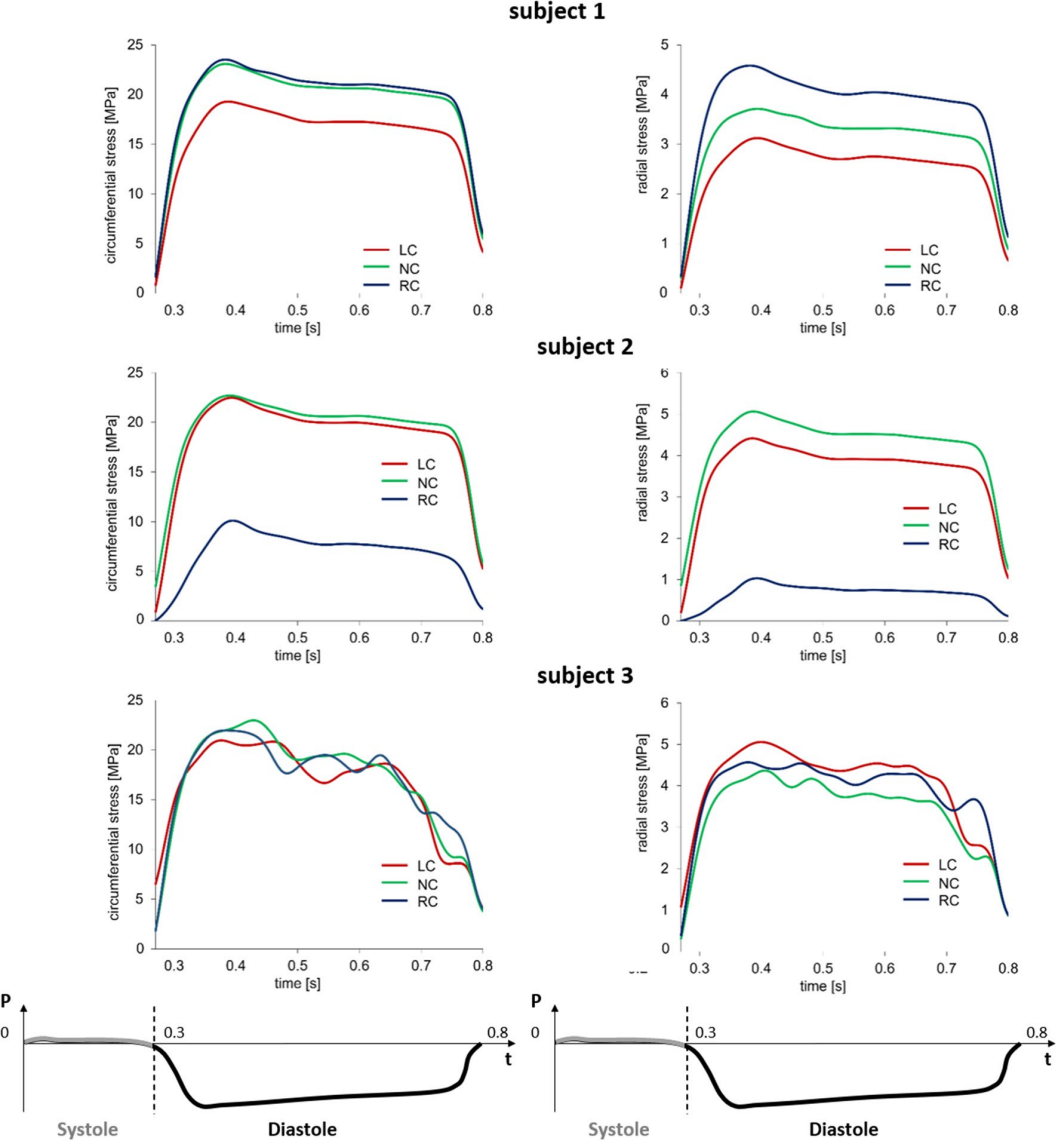
Circumferential (left column) and radial (right column) stress in the fibrosa layer of the patch for the LC, NC and RC leaflets over the diastolic phase of the 2nd cardiac cycle for the three subjects. The time-course of the transvalvular pressure is reported in the bottom row of each column

**Fig. 11:**
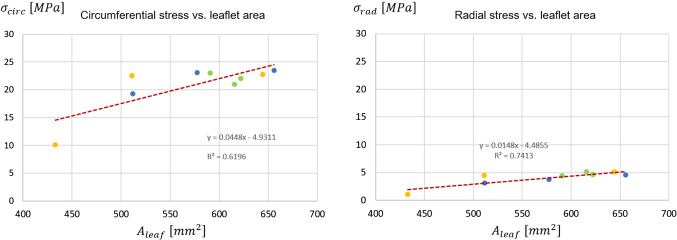
Correlation of circumferential stress ($\sigma _{circ}$) and radial stress ($\sigma _{rad}$) with the leaflet surface area. For each stress and strain component, the data represents the maximum value reported in the plots of Fig. 10. Dots = data computed by the models of subject 1 (blue), subject 2 (yellow) and subject 3 (green); dashed red line = regression line. $R^2$ is reported

Because of this evidence, and because the fibrosa layer is the most prone to undergo remodeling Yip and Simmons (2011), the stresses in the circumferential and radial direction were extracted from the pre-stressed patches just for this layer over the diastolic phase of the cardiac cycle (Fig. 10). Peak diastolic circumferential and radial stresses in the fibrosa ranged from 10.1 MPa to 23.5 MPa and from 1.0 MPa to 5.0 MPa, respectively. For each leaflet, these values were approximately 20 and 15 times greater, respectively, than the peak diastolic circumferential and radial stresses computed at the organ length-scale where leaflet tissue was described through a homogeneous equivalent. At this scale, it has to be remembered that the patches are pre-stretched in order to include the systolic phase deformation (as described in Sect. 2.3); this process impacts the stress curve generated as clearly visible for the RC leaflets. Also, correlations were checked for between each stress component of the peak diastolic stress in the fibrosa and the leaflet surface area (Fig. 11): linear positive correlations with $R^2$ equal to 0.62 and 0.74, respectively, were found.

### Cell length-scale model

**Fig. 12:**
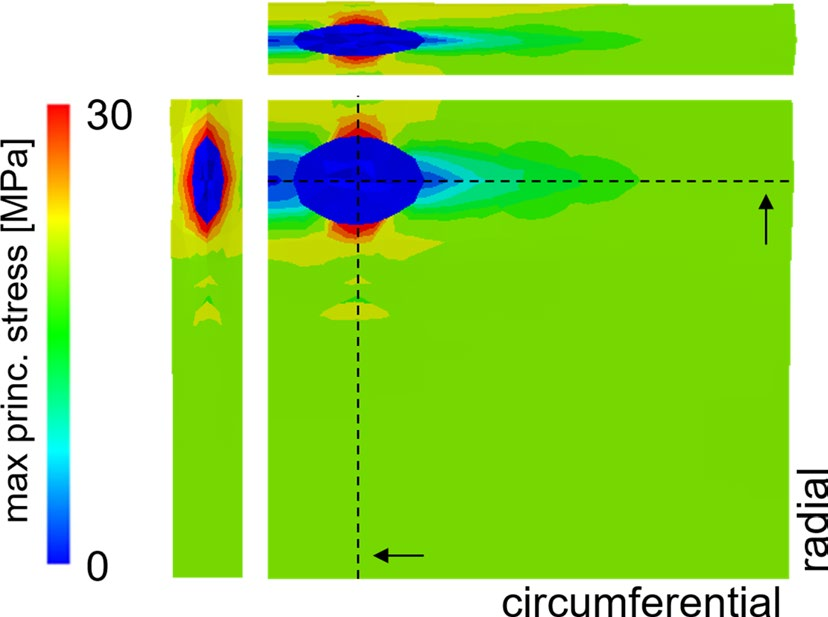
Max principal stress on the cell length-scale model. The sections on the circumferential and radial plane are plotted on the top and left of the patch, respectively.

Even if a complete mesh continuity exists between the VIC and the ECM, the discontinuity in mechanical proprieties between the two led to a strong and local stress increase at the ECM-VIC interface, where the cell itself acts as a stress raiser. This behavior is depicted in Fig. 12 for the LC leaflet of subject 1 to show the qualitative behavior observed in each cell-scale simulation, i.e., in every leaflet of every modeled subject.

**Fig. 13:**
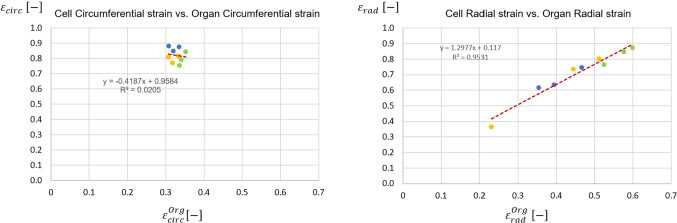
Linear regression between the leaflet and the cell strain in the circumferential (left-hand side) and radial direction (right-hand side). The corresponding $R^2$ is reported. Dots = data computed by the models of subject 1 (blue), subject 2 (yellow) and subject 3 (green); dashed red line = regression line

In Fig. 13, the linear regression between the max strain at the organ length-scale and the max strain at the cell length-scale is reported. The narrow range in the circumferential direction noticed at the organ scale (see Sect. 3.1), was found here for the cell scale, where the deformation ranges between 0.75 to 0.88. In the radial direction instead, as for the organ scale, the max strain range at the cell scale is wider, going from 0.36 to 0.87; the correlation between the organ scale deformations and the cell strain deformations gave a p-value $< 0.0001$ and $R^2 = 0.9521$, showing a high correlation between the two datasets.

## Discussion

*Novelty of the study* - In the present study, we developed a FE workflow for the multiscale analysis of AV biomechanics starting from subject-specific anatomies. The workflow was applied to three physiological subject anatomies with the aim of capturing the effect of subject-specific and leaflet-specific anatomical features at the organ scale down to the cell scale, in order to obtain a range of strain values describing the biomechanical milieu of VICs in physiological conditions. To the best of our knowledge this is the first AV model tackling the multiscale biomechanical analysis starting from organ length-scale models that combine anatomical realism based on clinical imaging with a state-of-the-art modeling of the anisotropic and hyperelastic stress-strain behavior of AV tissue. In our experience Votta et al. (2017) Sturla et al. (2016), combining these two features strongly impacts the computed leaflet strain and stress fields. Being able to account for the actual anatomical features of the aortic valve leaflets allowed to gain insight into their effect on the structural mechanics of the leaflets at all length-scales, down to the cell scale. In this perspective, the second element of novelty of our approach is relevant. This is represented by the mixed approach used to transfer organ-scale information down to the cell length-scale exploiting different BCs: displacement data from the organ-scale model were used to impose kinematic BCs to the tissue length-scale model, and stress data from the latter were used to impose loading BCs to the cell length-scale. Loading boundary conditions were applied on the external faces of the cell length-scale fibrosa patches as normal distributed loads upon verifying that shear loads were about two orders of magnitude smaller. This solution allowed us to exploit symmetry boundary conditions and limit the computational expense of the cell length-scale simulations. The application of displacement boundary conditions would have potentially required to impose displacement components both normal and tangential to the boundary surface. Tangential components would have been not always negligible; if accounted for, these would have prevented from the use of symmetry conditions, while if neglected these may have led to a cruder approximation

*Relevance of subject-specific geometry* - As previously mentioned, the three modeled aortic roots shared the same assumptions on loading conditions, tissue mechanical properties and tissue thickness. Hence, the comparison of the field variables computed for the various leaflets provide some insight into the influence of major anatomical differences, i.e., leaflet extent and shape, on leaflet strains and stresses. At the organ length-scale, peak radial leaflet strains well correlated with leaflet surface area ($R^{2}=0.67$); conversely, circumferential leaflet strains varied over a narrow range of values regardless of leaflet surface extent ($R^{2}=0.40$)(Fig. 8). The different dependency of the two strain component on the leaflet surface area can be explained based on the coaptation mechanisms in healthy aortic valves: during transient closure, each leaflet moves downwards and inwards, so that the central portion of the free margin reaches approximately the axis to the valve orifice. Once coaptation starts, the coaptation area of each leaflet extends to the region between the free margin and the leaflet belly; this region remains suspended with no major stretches owing to the contact interaction with the other leaflets. Concomitantly, the belly region of each leaflet is not directly sustained by this interaction and undergoes bending owing to the transvalvular pressure load. In the radial direction, this bending effect and the associated strain can be more marked in a larger leaflet, because there is no physical constraint to the downward (i.e., toward the ventricle) motion of the leaflet belly. Conversely, in the circumferential direction, this effect is prevented by the presence of physical constraints, i.e., the commissures that delimit each leaflet and the other leaflets of the closed valve. For the circumferential stretch to be larger in a larger leaflet, the latter should be able to extend more in the circumferential direction; this could occur only if the leaflet would displace its counterparts toward the respective sides of the aortic valve, i.e., if valve closure was not physiological. The circumferential and radial components of the peak diastolic stresses in the belly region had a different dependency on leaflet surface area as compared to the corresponding strain components; both stress components increased when larger leaflets were considered ($R^2 = 0.80$ and $R^2 = 0.67$ for the circumferential and radial component, respectively) (Fig. 8). The dependency was more pronounced for the circumferential component, as the slope of the corresponding regression line was twofold with respect to the one associated to the radial component. As a result, in larger leaflets, we did observe larger radial strains as well as larger radial stresses, and concomitantly we observed larger circumferential stresses but not larger circumferential strains. This combination of trends was common to each leaflet, regardless of the leaflet surface area and regardless of the proportions between the leaflet and the two other leaflets of the same valve. Such computational evidence is not an inconsistency and is a clear consequence of the anisotropic stress-strain behavior modeled for the valve leaflets, based on the assumption of the presence of collagen fibers embedded in an elastic isotropic matrix and preferentially oriented in the circumferential direction. We may hence infer that in a physiological aortic valve leaflet diastolic strains and stresses at the macroscale are largely influenced by the leaflet-specific anatomy, but the way strain and stress components are combined is dominated by tissue microstructure. The dependency of leaflet biomechanics on the leaflet-specific anatomy observed at the organ length-scale is reflected, and to some extent emphasized, into the results obtained at the lower length-scales. At the tissue length-scale, the peak diastolic circumferential and radial stresses computed in the fibrosa (Fig. 11) correlated with the leaflet surface area. As compared to the corresponding observations at the organ length-scale, similar values were obtained for the coefficient of determination of the linear regression lines. The slope of the respective regression lines for the two stress components was approximately 44.8 and 15.8 $\mathrm{kPa}/{\mathrm{mm}^{2}}$, whereas at the organ length-scale slopes equal to 2.1 and 1.1 $\mathrm{kPa}/{\mathrm{mm}^{2}}$ were found. Hence, along the circumferential and radial direction the slope increased by a factor of approximately 22 and 15, respectively, consistently with the higher stress levels associated to the much stiffer response of the fibrosa as compared to the homogeneous equivalent material used to describe leaflet tissue at the organ length-scale. At the cell length-scale, the difference between the strains in two main directions, and between the respective relationships with the specific leaflet anatomy, was even more evident than at the organ length-scale. Computed cell strains support the findings of the work published by Lewinsohn et al. (2011), where the authors suggest that the organization of the ECM within leaflet tissue acts to reduce direct tensile strain transfer to cells in the circumferential direction. Of note, thanks to the approach adopted to transfer information between length-scales, strain values obtained at the cell length-scale do not simply replicate the corresponding values at the organ length-scale as in the work of Weinberg and Mofrad (2007), but are shifted toward higher strain values. This finding suggests that VICs strains, especially in the radial direction, cannot be inferred directly from an organ length-scale strain analysis.

**Fig. 14:**
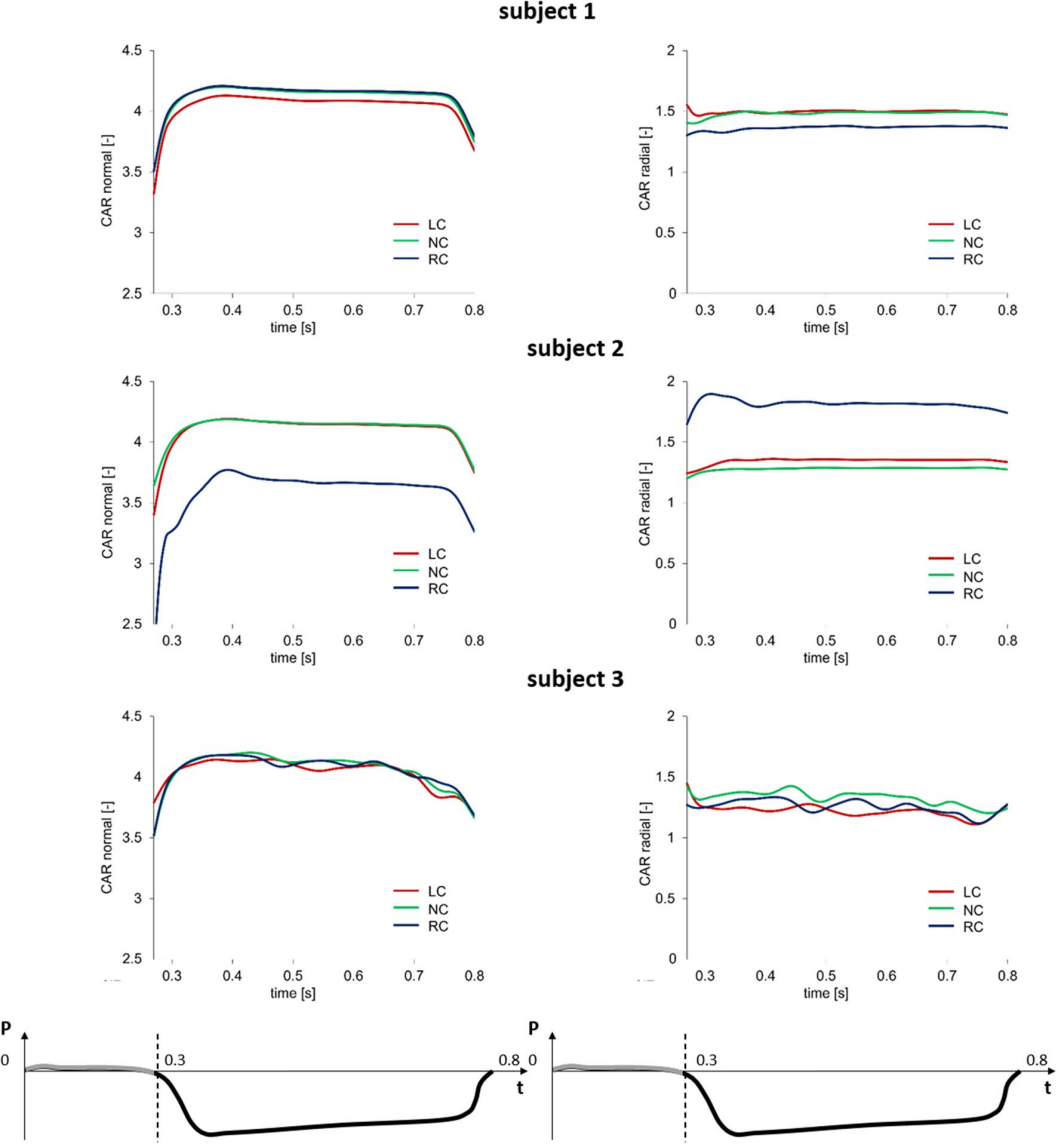
Normal (left column) and radial (right column) cell aspect ratio (CAR) in the fibrosa layer of the patch for the LC, NC and RC leaflets over the diastolic phase for the three subjects. The time-course of the transvalvular pressure is reported in the bottom row of each column

*Comparison of computational results vs. previous evidence* - Even if characterized by inter-leaflet variability, leaflet she strain curves computed during the cardiac cycle at the organ scale showed good agreement in terms of strain values with experimental data from Thubrikar on canine AVs Thubrikar (1979). However, systolic strains in the circumferential direction overestimated (by about $10\%$) the corresponding experimental measurements; this effect might be due to the choice of the leaflet region where these values were computed, which in our case represents the most deformed region of the entire leaflet. In fact, the highest strain values are concentrated in the thinner belly region, while the attachment edge showed almost no-deformation in both the circumferential and radial direction, matching the same strain pattern obtained by Joda et al. (2016) on a 3D FSI computational model of the AV including leaflet thickness inhomogeneity and material anisotropy. In the tissue-scale simulations, we provided an insight into the strongly in homogeneous stress distribution among the different AV leaflet layers. The fibrosa showed a load bearing role within the three AV layers, reflecting the higher concentration of type I aligned collagen fibers and in agreement with the stress trend in Rego and Sacks (2017); this behavior was not observable from the organ scale level, wherein leaflet tissue was modeled as homogeneous, and only a layer-dedicated mechanical description allowed for this type of investigation. Based on the cell-scale simulations, the cellular aspect ratio (CAR) of the VIC virtually seeded in the fibrosa was computed for the sake of comparing our data vs. those predicted in the work by Weinberg and Mofrad (2007). Despite being dependent on the specific leaflet considered (Fig. 14, Table 3), our CAR values well compare to the ones by Weinberg and Mofrad (2007), which were obtained from paradigmatic organ-scale models.

**Table 3:** Peak diastolic cellular aspect ratio (CAR) values computed for each leaflet. LC left coronary; NC non coronary, RC right coronary

Leaflet	Normal CAR	Radial CAR
*Subject 1*		
LC	4.1	1.5
NC	4.2	1.5
RC	4.2	1.4
*Subject 2*		
LC	4.2	1.4
NC	4.2	1.3
RC	3.7	1.4
*Subject 3*		
LC	4.2	1.3
NC	4.2	1.4
RC	3.1	1.3

In addition, the discontinuity in mechanical properties between the VIC and the fibrosa ECM is such that, as previously reported by Huang et al. (2007), cell-scale key results are insensitive to changes in VIC mechanical properties as long as the discontinuity remains.

*Limitations and future directions* - The most important limitation of this work is related to the assumptions made on leaflet thickness, which could not be assessed from cMRI images on a subject-specific basis. Hence, thickness values from literature were set for each leaflet of each subject. We may speculate that leaflet thickness could be related to leaflet surface extent: namely, we expect a thicker section for wider leaflets and a thinner section for smaller ones in order to compensate for the generation of deformation/stresses deviating from a homeostasis range. The thickness value represents therefore a key variable in the model definition, and our future technical efforts will be concentrated on changing the imaging technique in order to obtain subject-specific thickness data and consequently a more reliable organ scale geometry. A second limitation consists in the fact that in the organ length-scale and tissue length-scale simulations two different approaches were used to describe the stress-strain response of AV leaflet tissue, which was assumed homogeneous in the former case and layered in the latter one. As a result, an approximation is introduced when passing information between the two length-scales. Third , the mechanical response of aortic wall tissue (distal portion of the left ventricle outflow tract, Valsalva sinuses, interleaflets triangles and ascending aorta) was described as homogeneous, linear elastic and isotropic, while the real tissue has a hyperelastic and anisotropic stress-strain response, that varies depending on the considered wall region Gundiah et al. (2008). In principle, this assumption could impact on the mechanical response of the aortic wall. However, aortic wall nonlinearity and anisotropy are not as marked as in AV leaflets Choudhury et al. (2009); as a result, the inaccuracies associated to our simplifying assumption are reasonably limited. A fourth limitation of the study is the lack of comparison of the model vs. ground truth data acquired on the same subjects whose cMRI images were considered to feed the models. Namely, the diastolic configuration of the valve as computed by the organ length-scale models should have been compared vs. the diastolic configuration of the valve as reconstructed from medical imaging. Such comparison would not be a sound validation of the models in terms of reliability of the computed strain and stress field but would at least allow to assess if the overall loaded geometry of the aortic valve leaflets is correctly captured. Because the subjects considered in this work were healthy volunteers, they did not undergo a complete imaging protocol as for, e.g., patients affected by aortic valve stenosis or by aortic valve regurgitation. Hence, the only images at our disposal for validation purposes were cMRI images in diastole; unfortunately, their space resolution, contrast, and quality do not allow to reliably reconstruct the profile of the leaflets so to capture their compound curvature. This limitation could be bypassed if ultrasound data acquired in multiple cut-views, even if via transthoracic echocardiography, were available. The need for such data should be born in mind in the future development of the present study. Such future development should also aim to enrolling a larger cohort of subjects, so to not only strengthen the robustness of the approach, but also to give a more statistically relevant estimation of the physiological VICs strain range. Moreover, expanding the analysis to a group of pathological subjects, e.g., BAV patients, would be the natural development of the study toward assessing pathological deviations in VIC deformations. On the long run, strain data derived from computational analyses could be used to drive in vitro experiments on real VICs, e.g., by means of bioreactors or lab-on-chips, in order to verify the effect of the mechanical stimuli on VIC activity. This might pave the way to obtain new insights on the complex relationship between external mechanical stimuli and VICs remodeling activity in the context of AV degenerative pathologies.

## References

[CR1] Akram J, Zhongmin J, Axel H, Jon S (2016). Multiphysics simulation of the effect of leaflet thickness inhomogeneity and material anisotropy on the stress-strain distribution on the aortic valve. J Biomech.

[CR2] Bakhaty Ahmed A, Sanjay G, Mofrad Mohammad RK (2010). Consistent trilayer biomechanical modeling of aortic valve leaflet tissue. J Biomech.

[CR3] Billiar KL, Sacks MS (2000). Biaxial mechanical properties of the natural and glutaraldehyde treated aortic valve cusp-Part I: Experimental results. J Biomech Eng.

[CR4] Bryn B, Bo W, Guangjun W, Robbin B, Raj P, Patnaik Sourav S, Ryan BJ, Andrew C, Erin B-F, Williams Lakiesha N, Jun L (2015). On the Bending Properties of Porcine Mitral, Tricuspid, Aortic, and Pulmonary Valve Leaflets. J Long-Term Effects Med Implants.

[CR5] Conti Carlo A, Emiliano V, Della CA, Del VL, Ciro B, Maurizio C, Alberto R (2010). Dynamic finite element analysis of the aortic root from MRI-derived parameters. Med Eng Phys.

[CR6] David Merryman W, Inchan Y, Lukoff Howard D, Krueger Paula M, Farshid G, Hopkins Richard A, Sacks Michael S (2005). Correlation between heart valve interstitial cell stiffness and transvalvular pressure: implications for collagen biosynthesis. Am J Physiol Heart Circul Physiol.

[CR7] Elena A, Peter L (2017). A rock and a hard place chiseling away at the multiple mechanisms of aortic stenosis. Circulation.

[CR8] Francesco S, Mattia R, Mattia V, Annalisa D, Riccardo V, Georgia P-M, Gaetano B, Emiliano V, Alberto R (2016). Impact of different aortic valve calcification patterns on the outcome of transcatheter aortic valve implantation: a finite element study. J Biomech.

[CR9] Guccione JM, McCulloch AD, Waldman LK (1991). Passive material properties of intact ventricular myocardium determined from a cylindrical model. J Biomech Eng.

[CR10] Hao LC, Carruthers Christopher A, Salma A, Gorman Robert C, Gorman Joseph H, Sacks Michael S (2015). Quantification and simulation of layer-specific mitral valve interstitial cells deformation under physiological loading. J Theor Biol.

[CR11] Holzapfel Gerhard A, Gerhard S, Gasser Christian T, Peter R (2005). Determination of layer-specific mechanical properties of human coronary arteries with nonatherosclerotic intimal thickening and related constitutive modeling. Am J Physiol Heart Circulatory Physiol.

[CR12] Huang HYS (2004) Micromechanical simulations of heart valve tissues. Bioengineering

[CR13] Ioana PM, Stefan S, Peter P, Christian H (2018). A mathematical multiscale model of bone remodeling, accounting for pore space-specific mechanosensation. Bone.

[CR14] Jane Grande K, Cochran Richard P, Reinhall Per G, Kunzelma Karyn S (1998). Stress variations in the human aortic root and valve: the role of anatomic asymmetry. Ann Biomed Eng.

[CR15] Johnson Joshua E, Troy Karen L (2017). Validation of a new multiscale finite element analysis approach at the distal radius. Med Eng Phys.

[CR16] Labrosse Michel R, Keegan L, Beller Carsten J (2010). Structural analysis of the natural aortic valve in dynamics: from unpressurized to physiologically loaded. J Biomech.

[CR17] Lewinsohn AD, Anssari-Benham A, Lee DA, Taylor PM, Chester AH, Yacoub MH, Screen HRC (2011). Anisotropic strain transfer through the aortic valve and its relevance to the cellular mechanical environment. Proc Inst Mech Eng Part H J Eng Med.

[CR18] Michele M, Giuseppe V (2012). Multiscale elastic models of collagen bio-structures: from cross-linked molecules to soft tissues. Stud Mechanobiol Tissue Eng Biomater.

[CR19] Namrata G, Kimberly K, Matthews Peter B, Julius G, Dwyer Harry A, David S, Chuter Timothy AM, Sloane Guy T, Ratcliffe Mark B, Tseng Elaine E (2008). Asymmetric mechanical properties of porcine aortic sinuses. Ann Thoracic Surgery.

[CR20] Nusrat C, Olivier B, Leonie R, Dominique T, Raymond C, Jagdish B, Rosaire M, Leask Richard L (2009). Local mechanical and structural properties of healthy and diseased human ascending aorta tissue. Cardiovascular Pathol.

[CR21] Otto CM (2002). Calcification of bicuspid aortic valves. Heart.

[CR22] Rego Bruno V, Sacks Michael S (2017). A functionally graded material model for the transmural stress distribution of the aortic valve leaflet. J Biomech.

[CR23] Sacks Michael S, Yoganathan Ajit P (2007). Heart valve function: a biomechanical perspective. Philos Trans Royal Soc B Biol Sci.

[CR24] Shadow HHY, Jun L, Sacks Michael S (2007). In-situ deformation of the aortic valve interstitial cell nucleus under diastolic loading. J Biomech Eng.

[CR25] Stella John A, Sacks Michael S (2007). On the biaxial mechanical properties of the layers of the aortic valve leaflet. J Biomech Eng.

[CR26] Taylor Patricia M, Puspa B, Brand Nigel J, Thomas Penny S, Yacoub Magdi H (2003). The cardiac valve interstitial cell. Int J Biochem Cell Biol.

[CR27] Thubrikar M (1979). The aortic valve.

[CR28] Vaughan TJ, McCarthy CT, McNamara LM (2012). A three-scale finite element investigation into the effects of tissue mineralisation and lamellar organisation in human cortical and trabecular bone. J Mech Behav Biomed Mater.

[CR29] Votta E, Presicce M, Della Corte A, Dellegrottaglie S, Bancone C, Sturla F, Redaelli A (2017). A novel approach to the quantification of aortic root in vivo structural mechanics. Int J Numer Methods Biomed Eng.

[CR30] Weinberg Eli J, Kaazempur MMR (2007). Three-dimensional, multiscale simulations of the human aortic valve. Cardiovascular Eng.

[CR31] Weinberg EJ, Shahmirzadi D, Mohammad MR, Kaazempur M (2010). On the multiscale modeling of heart valve biomechanics in health and disease. Biomech Model Mechanobiol.

[CR32] Yin YCY, Craig S (2011). The aortic valve microenvironment and its role in calcific aortic valve disease. Cardiovascular Pathol.

[CR33] Yusuke S, Buchanan Rachel M, Johannah S-A, Farshid G, Sacks Michael S (2017). On the functional role of valve interstitial cell stress fibers: a continuum modeling approach. J Biomech Eng.

